# E3 ubiquitin ligase Nedd4 inhibits AP-1 activity and TNF-α production through targeting p38α for polyubiquitination and subsequent degradation

**DOI:** 10.1038/s41598-017-04072-2

**Published:** 2017-07-03

**Authors:** Qingjun Liu, Shihui Zhang, Gan Chen, Hong Zhou

**Affiliations:** Beijing Institute of Transfusion Medicine, Beijing Key Laboratory of Blood Safety and Supply Technologies, Taiping Road 27, 100850 Beijing, P.R. China

## Abstract

p38α plays an important role in many inflammatory diseases, such as skin inflammation, endotoxic shock and arthritis. Ubiquitination is a vital posttranslational modification of proteins and plays a crucial regulatory role in inflammatory cells. It has been reported that ubiquitination of Tak1 and TAB1 upstream of p38α can regulate p38α activation respectively. However, p38α ubiquitination is not yet clear. In this paper, we showed that E3 ubiquitin ligase Nedd4 is a regulatory component of the p38α pathway and is responsible for polyubiquitination of p38α through K48-linked and K63-linked polyubiquitination. The levels of p38α and its downstream target TNF-α were increased in Nedd4 deficient macrophages response to LPS compared with wild-type cells. AP-1 activity and degradation of p38α were induced by Nedd4 in a dose-dependent manner. Furthermore, we found that phosphorylation of p38α is involved in the interactions between p38α and Nedd4 and subsequently promotes polyubiquitination of p38α, especially K48-linked polyubiquitination by Nedd4. The different conformation of two p38α isoforms (p38αV1 and p38αV2) might be the cause of their different interactions with Nedd4 and their polyubiquitination sites by Nedd4. Thus, NEDD4 is a previously unknown component of the p38α signaling complex necessary for TNF-α activation.

## Introduction

The mitogen-activated protein kinase (MAPK) p38α plays a crucial role in inflammatory signals derived from cytokine receptors or in response to infections^1^. p38 was phosphorylated by MKK3 (MAPK kinase kinase 3) and MKK6, thus mediating MAPK pathway^1^. The activation of p38α is associated with inflammatory skin disorders, including hapten-induced contact dermatitis, ultraviolet (UV) irradiation–induced dermatitis, and human psoriatic lesions^2–6^. p38α ablation targeted to the epithelial compartment is sufficient to suppress UVB-induced inflammation^6^. Myeloid-specific (but not skin epithelium–specific) deletion of the gene encoding p38α protects mice from experimentally induced skin inflammation, which suggests a cell specific role for p38α in skin inflammation^7^. The conditional deletion of Mapk14 (encoding p38α) in macrophages reduces the TLR-mediated induction of TNF-α and renders mice more resistant to endotoxic shock^8^. The effects of p38 inhibitors on TLR-induced TNF production, as well as their protective effect in antibody transfer models of arthritis, are due to the inhibition of p38α rather than the inhibition of p38β^9^. However, no p38α inhibitors are currently available that have progressed beyond phase 2 clinical trials because of toxicity and the appearance of bypass or escape mechanisms though the crucial role of p38α in regulating the inflammatory response^10, 11^. Thus, specific manipulation based on the physically regulatory mechanism of p38α will be an alternative pathway inhibiting p38α activation.

Posttranslational modifications of proteins that are mediated by ubiquitin conjugation play a crucial regulatory role in inflammatory cells^12, 13^. Ubiquitination involves a cascade of biochemical reactions through ubiquitin activating enzymes (E1), ubiquitin-conjugating enzymes (E2), and ubiquitin ligase (E3). Although ubiquitin contains seven lysine residues, its linkage to adjacent ubiquitin molecules in a ubiquitin chain generally occurs through either lysine 48 (Lys48, K48) or lysine 63 (Lys63, K63). K48-linked polyubiquitination predominantly targets proteins for proteasomal or lysosomal degradation, whereas K63-linked polyubiquitination results in non-proteasomal modifications, such as changes in subcellular localization or protein-protein interactions. The E3 ubiquitin ligases are critical components of this system, because they recognize, bind to, and recruit specific target proteins for ubiquitination^14^.

Nedd4 is an E3 ubiquitin ligase that belongs to the HECT (homologous to the E6-AP C terminus) family. Nedd4 contains an N-terminal C2 domain, three or four WW domains, each of which contains two conserved tryptophan residues, and the HECT ligase domain^15^. The C2 domain is a calcium dependent lipid-binding domain around 116 amino acids in length that targets proteins to phospholipid membrane^16^ and is also involved in protein-protein interactions^17^. The WW domains mediate protein-protein interactions by interacting with proline rich PPxY motifs and can also interact with phosphor-serine/threonine residues in substrates^18^. Many Nedd4 substrates have been identified by a number of *in vitro* studies and proteomic approaches so far^15^. The HECT domain contains a conserved cysteine that forms an intermediate thioester bond with the activated ubiquitin accepted from an E2 before catalyzing the ubiquitination of a lysine in the substrate protein^19^. Mice deficient in Nedd4 (Nedd4^−/−^ mice) were neonatal lethal, with delayed embryonic development and reduced growth and body weight due^20^. It has been found that Nedd4 plays an important role in immune cells including T cells^21^ and B cells^22^, however, the physiological functions and molecular mechanism of Nedd4 in macrophages still need to be investigated.

In this paper, we demonstrated that the E3 ubiquitin ligase Nedd4 regulates TNF-α expression by inhibiting the activation of p38α through K48 and K63-linked polyubiquitination of p38α. Phosphorylation of p38α is involved in the interaction between p38α and Nedd4 and subsequently promotes polyubiquitination of p38α, especially K48-linked polyubiquitination by Nedd4. The different association of p38α isoforms (p38αV1 and p38αV2) with Nedd4 and their different polyubiquitination sites by Nedd4 might result from the different conformation of p38αV1 and p38αV2. Nedd4 induces degradation of p38α in a dose-dependent manner. The interactions between Nedd4 and p38α might be used therapeutically to treat inflammation.

## Results

### p38α, especially phosphorylated p38α, was enhanced in Nedd4 knockdown and knockout macrophages response to LPS stimulation

**Figure 1:**
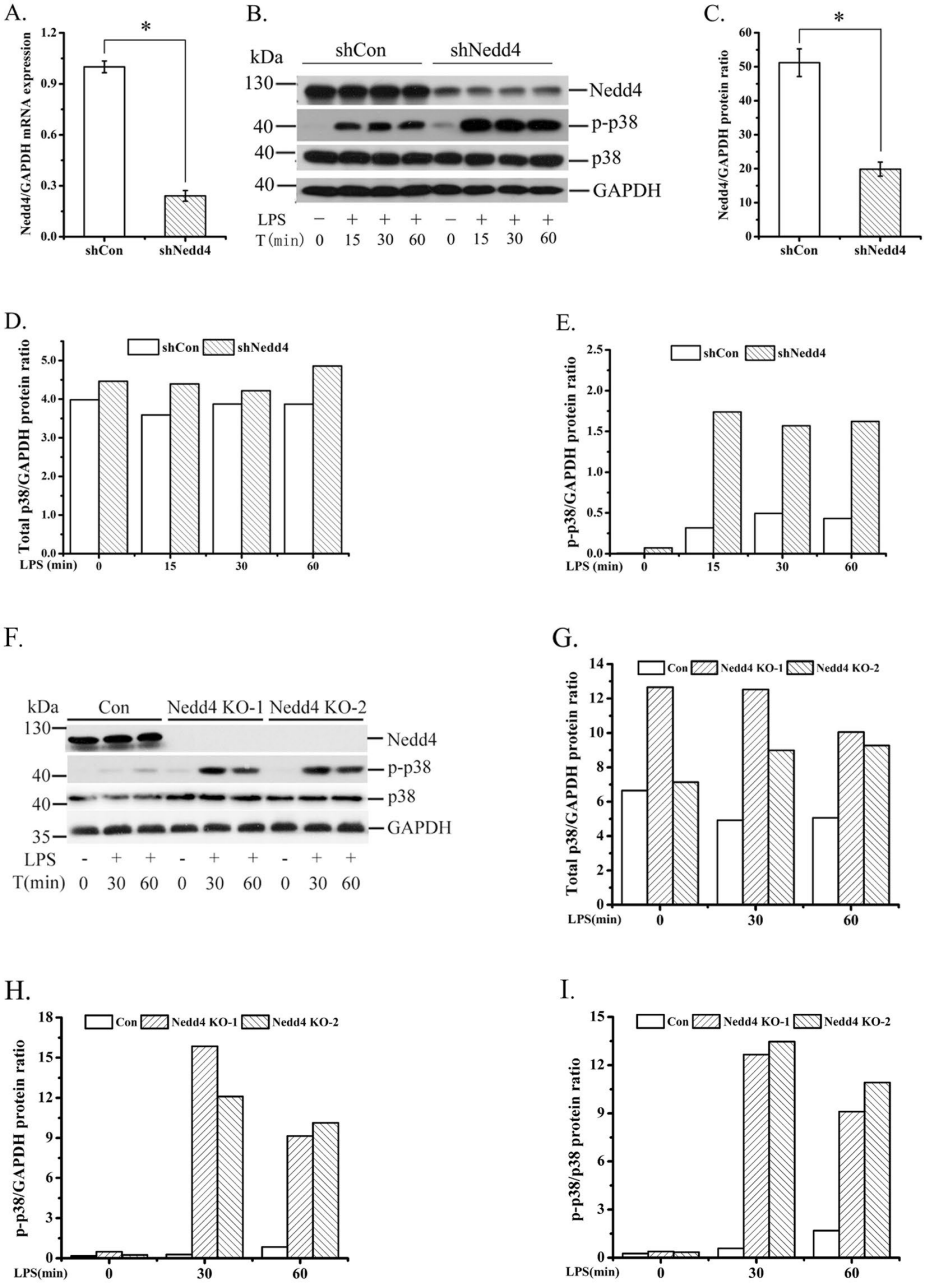
Nedd4 deficiency enhances p38α, especially p-p38αprotein levels. (**A**, **C**). mRNA and protein level of Nedd4 are decreased in iBMDM cells with lentiviral-based shRNA. (**A**) iBMDM cells transduced with control or shRNA targeting to Nedd4 were collected and the total RNA was extracted with TRIzol, reverse transcribed, and analyzed for Nedd4 mRNA with Q-PCR. (**B**, **F**). The p38, especially p-p38 protein levels were increased in Nedd4 deficient iBMDM cells stimulated for 0-60 min (above lanes) with LPS. (**B**) iBMDM cells transduced with control or shRNA targeting to Nedd4 were subjected to immunoblot analysis. (**F**) Immunoblot analysis of Nedd4 knockout (Nedd4^−/−^) iBMDM cell lines. (**C**–**E**). ImageJ analysis for the immunoblot of Nedd4, total p38 and p-p38 levels in iBMDM cells transduced with control or shRNA targeting to Nedd4. (**G**–**I**) ImageJ analysis for the immunoblot of total p38 and p-p38 levels in two Nedd4^−/−^ iBMDM cell lines. The results are represented from at least three independent experiments.

As the roles of Nedd4 in macrophages are elusive, we began by establishing Nedd4-silencing iBMDM cells with shRNA specific to Nedd4 to investigate the functions of Nedd4 in macrophage. The knockdown efficiency of Nedd4 in iBMDM cells was about 76% at the mRNA level (Fig. 1A) and 62% at the protein level (Fig. 1C). Then, we stimulated cells with LPS at different time points (0, 15, 30, 60 min). We found that the levels of phosphorylated p38 (p-p38) in Nedd4 knockdown cells at these time points were significantly increased compared with controls (Fig. 1B,E) and the levels of total p38 were also increased but not significantly compared with controls (Fig. 1B and D). To further confirm the results that Nedd4-silencing was not due to off-target effect of RNA interference (RNAi), we established Nedd4^−/−^ iBMDM cells with CRISPR/Cas9 method. We designed sgRNA sequence specific to exon1 of Nedd4 and subsequently inserted it into PX330 vector. There was deletion of 16 bases at Cas9 cleavage site which resulted in frame shift of Nedd4 (Supplementary Fig. 1). The blotting band of Nedd4 protein in one iBMDM clone completely disappeared (Supplementary Fig. 1B). The levels of p-p38 in Nedd4^−/−^ iBMDM responsive to LPS stimulation at 0 and 15 min were also markedly increased compared with controls (Supplementary Fig. 1B), though there was no further increase in Nedd4^−/−^ iBMDM at 60 min. Furthermore, we established two other Nedd4^−/−^ iBMDM cell lines (sgRNA sequences specific to exon1 and exon4 of Nedd4 respectively) using different methods. The levels of p38, especially p-p38 were also enhanced in these two Nedd4^−/−^ iBMDM cell lines stimulated with LPS at different time points (0, 30, 60 min) (Fig. 1F,G,H and I).

There are four p38MAPKs isoforms (p38α, p38β, p38γ, p38δ) which are encoded by different genes and have different tissue expression patterns. The p38α is ubiquitously expressed at significant levels in most cell types, whereas the others seem to be expressed in a more tissue-specific manner: p38β in the brain, p38γ in skeletal muscle and p38δ in endocrine glands^23^. In macrophages, p38α and p38δ are abundant, but p38β and p38γ are not detectable^24^. However, the anti-p38 IgG and anti-p-p38-IgG we used could detect p38α, p38β and p38γ, but not p38δ. Thus the p-p38 and total p38 in iBMDM cells we detected should respectively be p-p38α and total p38α using anti-p-p38 IgG and anti-p38 IgG. The above results indicated that the expression of Nedd4 is essential for regulating LPS-mediated activation of the p38α.

### p38α, especially p-p38α might be involved in Nedd4 ubiquitination system

**Figure 2:**
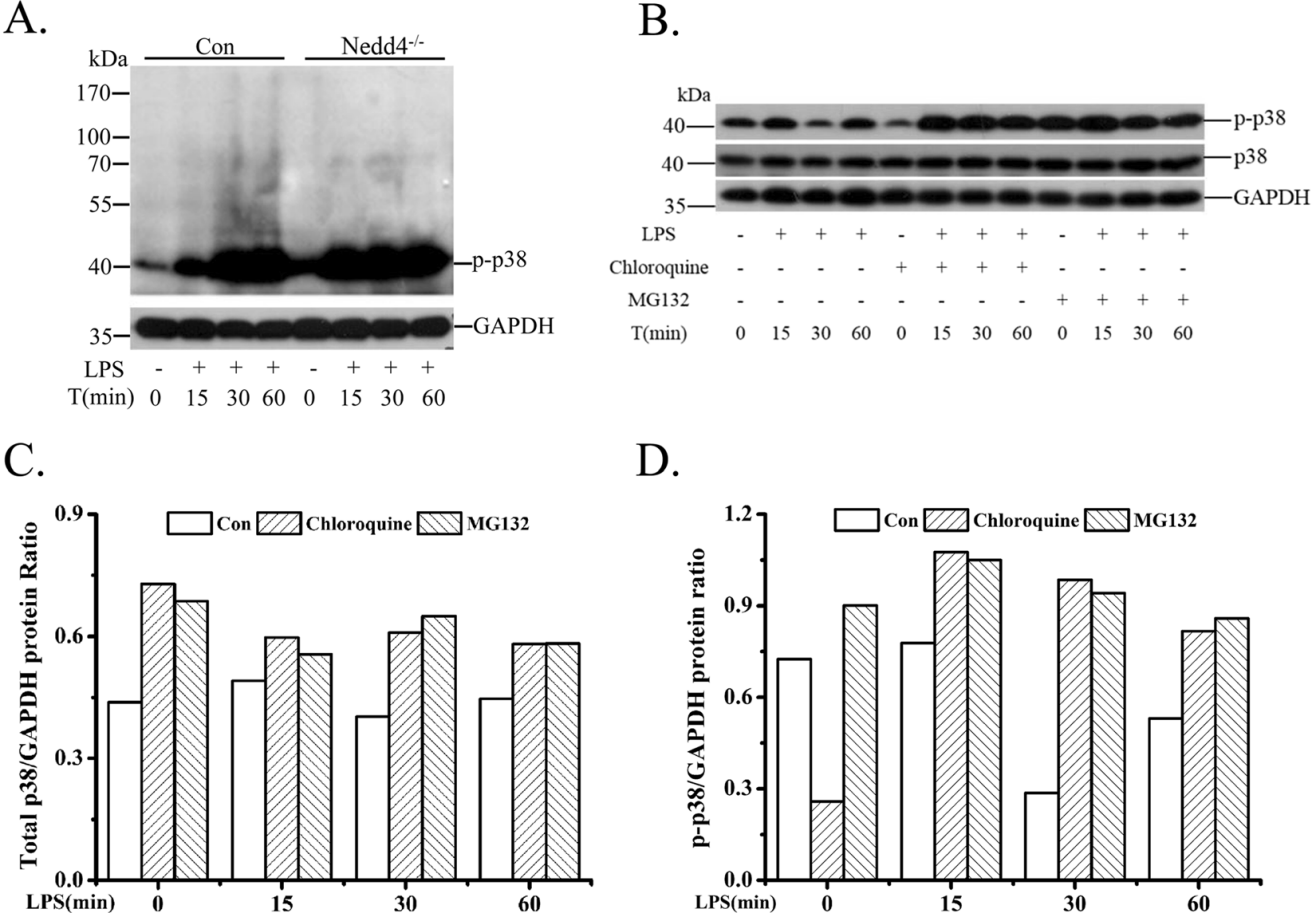
p38, especially p-p38α might be involved in Nedd4 ubiquitination system. (**A**) The smear bands above 40 kDa almost disappeared in Nedd4^−/−^ iBMDM cells. Overexposure for immunoblot analysis of p-p38 in wild type and Nedd4^−/−^ iBMDM cells stimulated for 0–60 min (above lanes) with LPS treatment. (**B**) p-p38 was degraded through lysosomal and proteasomal pathways. Immunoblot analysis of p-p38 in wild-type iBMDM cells stimulated for 0–60 min (above lanes) with LPS stimulation and Chloroquine or MG132 respectively. The results are represented from at least three independent experiments. (**C**,**D**) ImageJ analysis for the immunoblot of total p38 (**C**) and p-p38 (**D**) levels in iBMDM cells treated with Chloroquine or MG132 respectively.

Interestingly, there were apparent smear bands of p-p38 greater than 40 kDa in the lysates of Nedd4 wild-type iBMDM cells compared with that of Nedd4^−/−^ iBMDM cells (Fig. 2A) when the membrane of Blot (Supplementary Fig. 1B) was exposed longer. Furthermore, the intensities of the smear bands were gradually enhanced following LPS stimulation time (Fig. 2A). As Nedd4 is one of E3 ubiquitin ligase, we speculated that Nedd4 might be responsible for the polyubiquitination of phosphorylated p38α. It has been demonstrated that ubiquitination-mediated protein degradation is mediated via the proteasomal and lysosomal pathways. In general, K48-linked polyubiquitination predominantly targets proteins for proteasomal or lysosomal degradation, meanwhile, K63-linked polyubiquitination sometime targets proteins for lysosomal degradation^25^. To determine the pathway of p-p38α degradation, we used inhibitors of the proteasomal (MG132) or lysosomal (Chloroquine) pathways to assess the degradation route for p-p38 in iBMDM cells. The results (Fig. 2B and D) showed that the levels of p-p38 were significantly increased when there were MG132 or Chloroquine, indicating that p-p38 was degraded through proteasomal and lysosomal pathways. Furthermore, the levels of total p38 were also increased, but not significantly in iBMDM cells treated with MG132 or Chloroquine (Fig. 2C). However, we found that the abundances of p-p38 were gradually decreased following LPS treatment (Fig. 2B and D), which might be resulted from the dephosphorylation of phosphatases. Above data suggested that the degradation of p-p38 through proteasomal and lysosomal pathways might result from the K48 and K63-linked polyubiquitination of p38α by Nedd4.

### Nedd4 inhibits TNF-α production and AP-1 activation mediated by p38α activation

**Figure 3:**
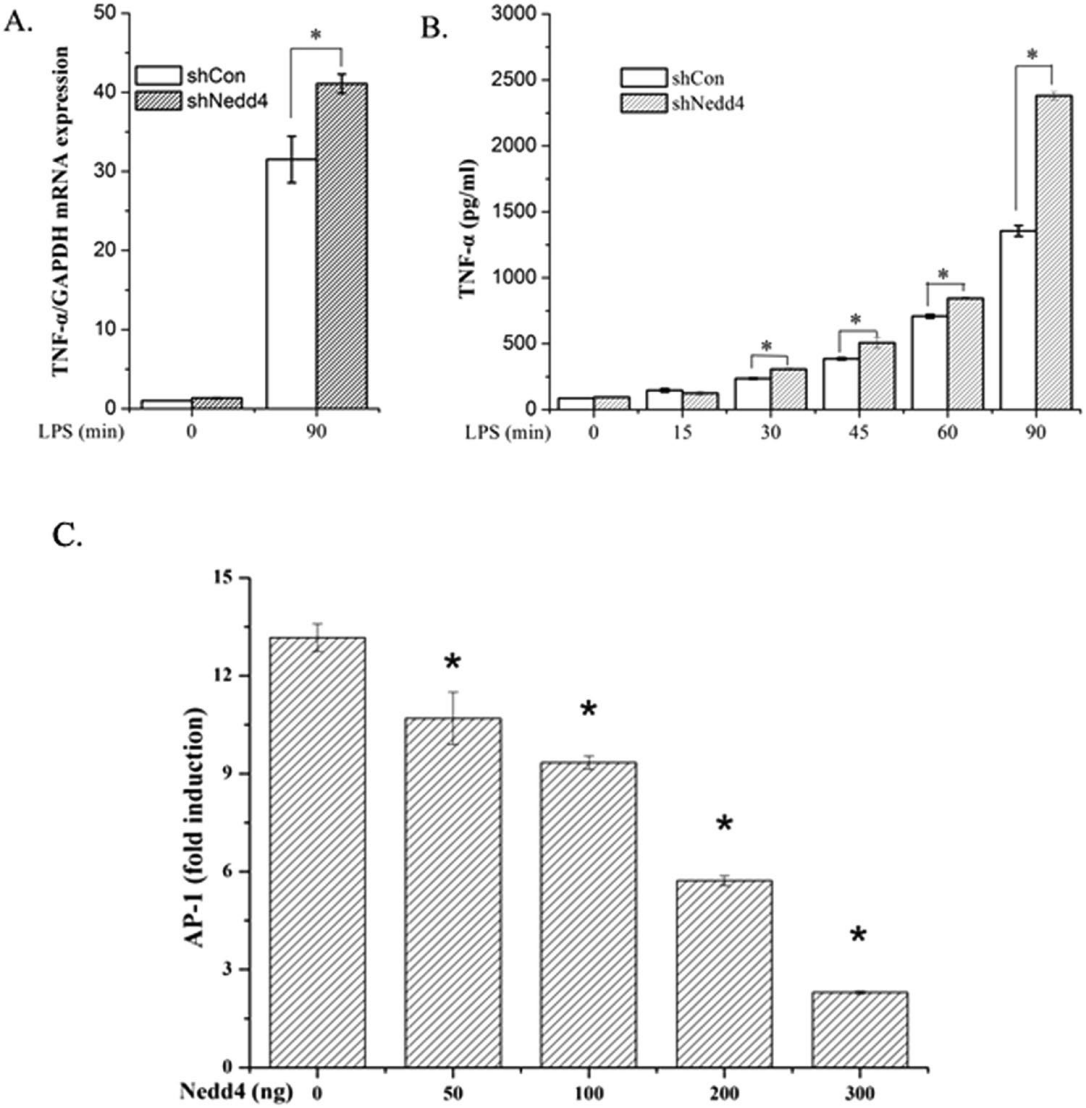
Nedd4 inhibits TNF-α production and AP-1 activation mediated by p38α activation. (**A**) mRNA level of TNF-α was enhanced compared with control cells. Control and Nedd4-silenced iBMDM cells were stimulated with LPS for 90 min, and total RNA was extracted. After reverse transcription, TNF-α mRNA was analyzed with Q-PCR. (**B**) ELISA of TNF-α in supernatants from control and Nedd4-silenced iBMDM cells stimulated for 0–90 min (above lanes) with LPS. Data are representative of three independent experiments with similar results (mean ± s.d.) (*, significant difference). (**C**) Nedd4 inhibits MyD88-dependent transcription of gene encoding AP-1. Luciferase assay of the induction of gene encoding AP-1 in lysates of 293T cells transfected with 150 ng pGL3-AP-1 (firefly-luciferase) reporter plasmid and 3 ng pGL3-TK (renilla-luciferase) reporter, and 50 ng MyD88-expressing plasmid plus 0, 50, 100, 200 or 300 ng Nedd4-expressing plasmid and cultured for 24 h. Empty control vector pcDNA3.1(+) was added to each sample to ensure transfection of the same amount of DNA in each. Luciferase activity is normalized to renilla luciferase activity and is presented relative to basal luciferase activity. *, p < 0.05 compared with no Nedd4 group. Data are the mean ± s.d. of 4 samples in one experiment representative of similar results obtained in three independent experiments.

In case Nedd4 is responsible for the ubiquitination of p-p38α and its subsequent degradation, Nedd4 will affect TNF-α and AP-1, which are the downstream targets of p38α in LPS-p38α signaling pathway. To confirm our hypothesis, we investigated the role of Nedd4 on TNF-α expression in Nedd4 knockdown iBMDM cells. In Nedd4-silenced iBMDM cells, TNF-α mRNA production in response to LPS was increased (Fig. 3A). The production of cytokine TNF-α by iBMDM-shNedd4 response to LPS, which was detected by ELISA, was increased compared with controls in response to LPS (Fig. 3B). The transcription factor AP-1 is the mediator between p38α and TNF-α, thus the effect of Nedd4 on p38α should interfere with AP-1 activation. As our speculation, the luciferase activity of AP-1 was inhibited by Nedd4 in dose-dependent manner (Fig. 3C). These results demonstrated that Nedd4 may indirectly regulate TNF-α expression and AP-1 activation through the degradation of p-p38α ubiquitinated by Nedd4.

### Nedd4 interacts with p-p38αV1 and p38αV2 by its WW domains

**Figure 4:**
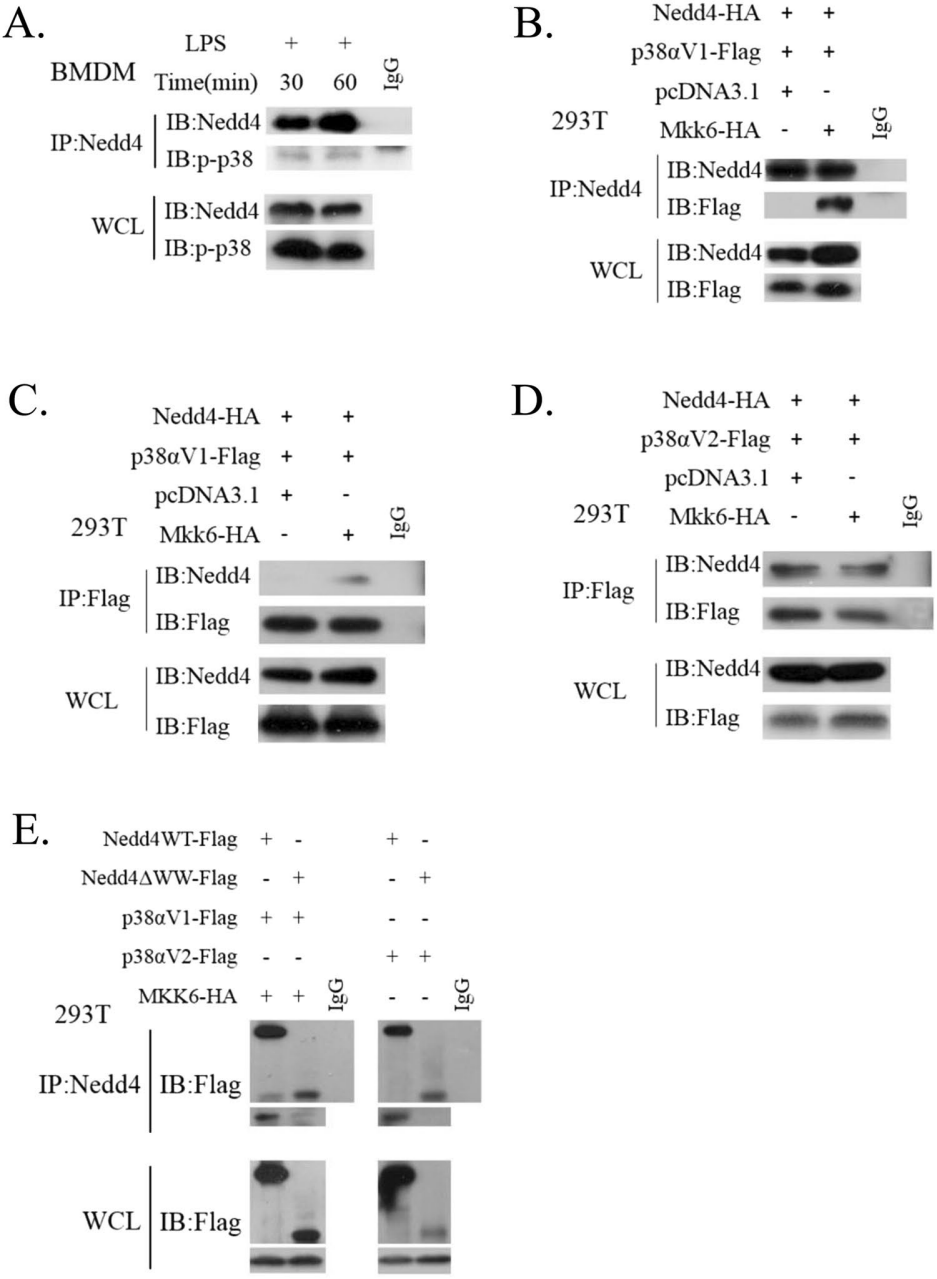
Nedd4 can interact with endogenous and exogenous p38α. (**A**) Nedd4 interacts with endogenous p-p38α. iBMDMs cells were incubated with LPS at the indicated times (30 min and 60 min), lysed, immunoprecipitated with anti-Nedd4, gel separated and detected with anti-p-p38 antibody. (**B**,**C**) The interaction between Nedd4 and p38αV1 requires phosphorylation of p38αV1 by MKK6. HEK293 cells co-transfected with various molecules (above lanes), lysed, immunoprecipitated with anti-Nedd4, gel separated and detected with anti-Flag antibody (**B**); immunoprecipitated with anti-Flag, gel separated and detected with anti-Nedd4 antibody (**C**). (**D**) The interaction between Nedd4 and p38αV2 does not require phosphorylation of p38αV2 by MKK6. 293T cells co-transfected with various molecules (above lanes), lysed, immunoprecipitated with anti-Flag, gel separated and detected with anti-Nedd4 antibody. (**E**) Nedd4 interacts with p-p38αV1 and p38αV2 by its WW domains. These results are represented from three independent experiments.

As mentioned above, p-p38α were enhanced in Nedd4 deficiency iBMDMs cells by LPS stimulation. Thus we speculated that p38α might interact with Nedd4. First of all, we found that there was a PPaY motif which was recognized by HECT E3 ligase in the protein sequence of p38α isoform2 (or variant 2, we called it as p38αV2) of C57/B6 mice in protein database and human p38α isoform1 in protein database of NCBI (http://www.ncbi.nlm.nih.gov/protein/) (Supplementary Fig. 2). Thus we hypothesized that p38α might be a direct substrate of NEDD4 E3 ligase activity and that p38α polyubiquitination by NEDD4 is a negative step for LPS-induced p38α activation. To test this hypothesis, we used immunoprecipitation experiments (IP) in macrophages. We found that NEDD4 physically interacted with p-p38α in iBMDMs cells treated with LPS at 30 min and 60 min (Fig. 4A).

To further confirm the interactions between Nedd4 and p38α, we established pcDNA(+)-p38α expression vectors by cloning p38α genes from cDNA of iBMDMs which added flag epitope at C-terminal of p38α by overlapping PCR. Though there were four variants of p38α in NCBI nucleotide database, both Variant 3 and Variant 4 coded small p38α proteins which deletes N terminal of p38α, thus we obtained p38αV1, p38αV2 clones in the next experiments. Then, we detected interactions between Nedd4 and p38α through co-IP from 293 T cells co-transfected with HA-Nedd4 and p38αV1-Flag or p38αV2-Flag respectively. We found that Nedd4 could interact with p38αV2 (Fig. 4D). However, we did not detect any interaction between Nedd4 and p38αV1 by Co-IP (Fig. 4B and C). As reported, MKK6 mediated phosphorylation of p38α, thus we speculated that phosphorylation of p38α might affect interactions between Nedd4 and p38α. To find out whether the interaction between Nedd4 and p38α necessitated phosphorylation of p38α, especially with p38αV1, we co-transfected MKK6 with HA-Nedd4 and p38α-Flag into 293T cells. The results showed that phosphorylation of p38αV1 by MKK6 promoted the interaction of Nedd4 with p38αV1 (Fig. 4B and C). However, such interaction were different from that between Nedd4 and p38αV2 whether there was MKK6 or not (Fig. 4D). These results indicated that Nedd4 could interact with p-p38αV1 and p38αV2.

To further determine whether the WW domains of Nedd4 mediate interactions between Nedd4 and p38α isoforms, we detected interactions between Nedd4△WW (Nedd4 mutant, whose WW domains are deleted)^26^ and p38α isoforms through co-IP from 293T cells transfected with HA-Nedd4△WW and p38αV1-Flag or p38αV2-Flag respectively. The results (Fig. 4E) showed that Nedd4△WW could not interact with p-p38αV1 and p38αV2, indicating that Nedd4 interacts with p-p38αV1 and p38αV2 by its WW domains.

### Nedd4 mediates K48/K63-linked polyubiquitination of p38αV1 and p38αV2, and phosphorylation promotes Nedd4 mediated K48-linked polyubiquitination rather than K63-linked polyubiquitination

**Figure 5:**
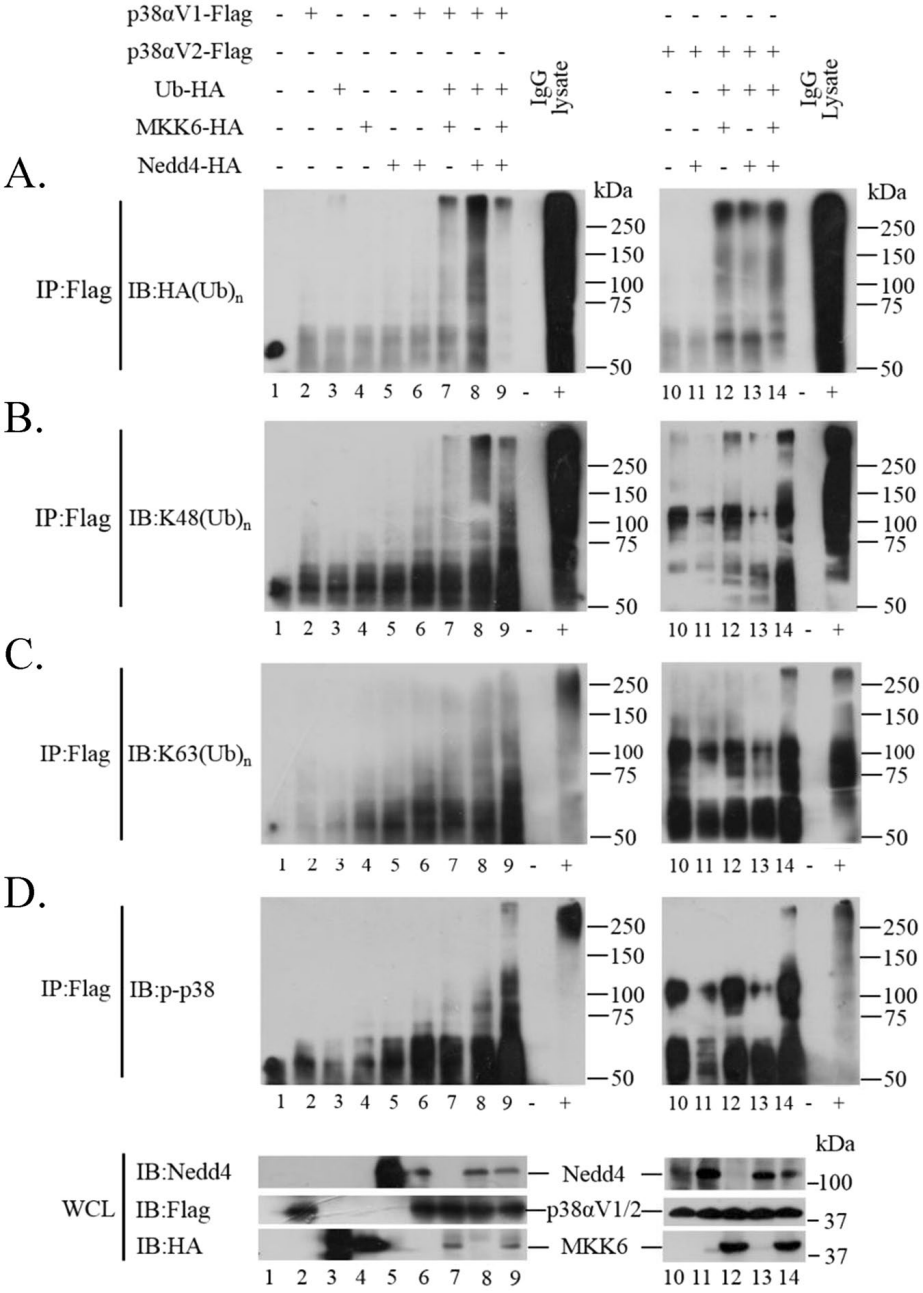
Nedd4 mediates polyubiquitination of p38α isoforms (Left. Ubiquitination of p38αV1; Right. Ubiquitination of p38αV2). Immunoblot analysis of anti-Flag immunoprecipitates of lysates of 293T cells co-transfected with various molecules (above lanes), probed with anti-HA (**A**), anti-K48 ubiquitin (**B**), anti-K63 ubiquitin (**C**) and anti-p-p38. For whole cell lysates, p38αV1 and p38αV2 were probed with anti-Flag, MKK6 was probed with anti-HA and Nedd4 was probed with anti-Nedd4. Lane 3 just showed the signals of ubiquitinated proteins, not MKK6.

**Figure 6:**
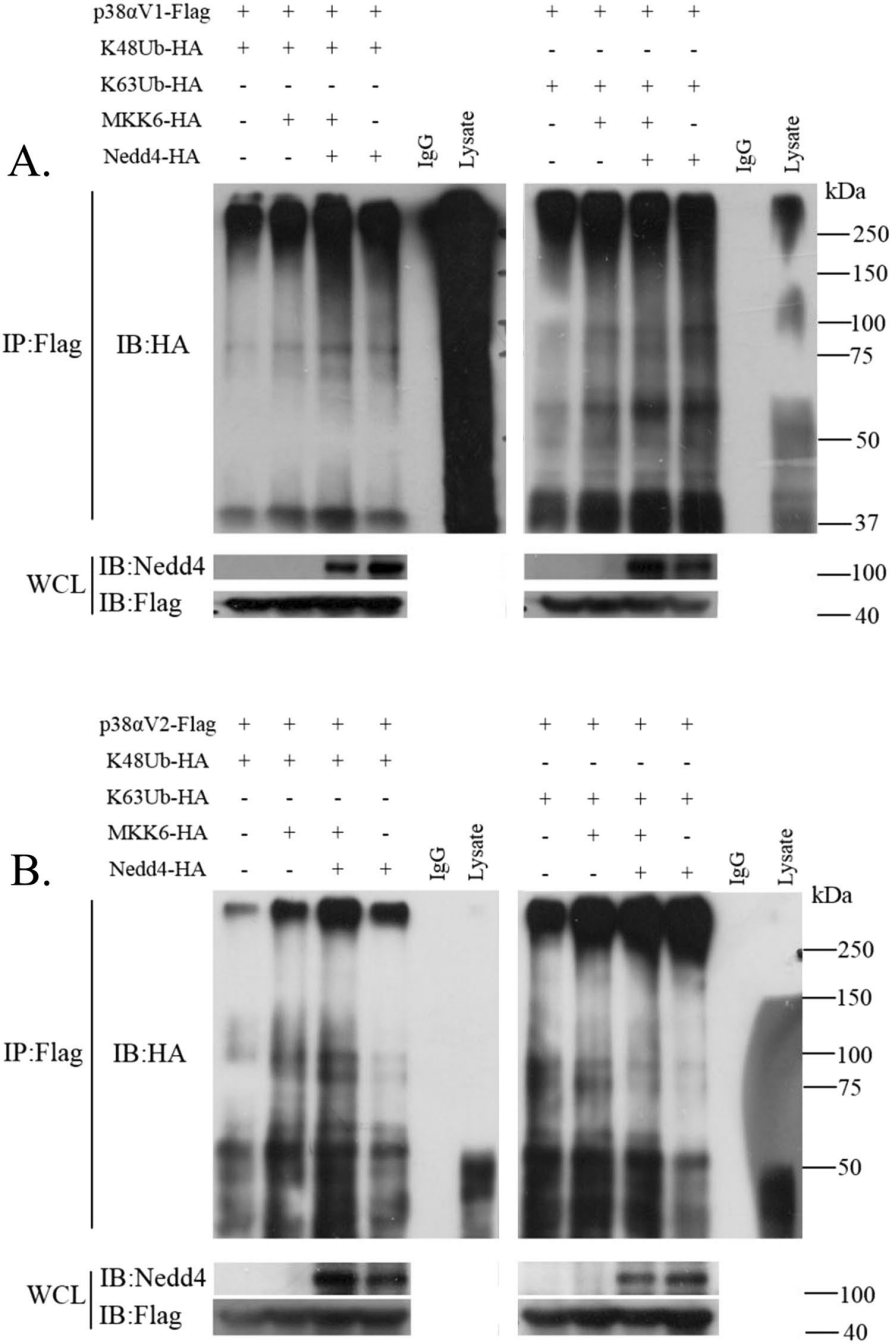
Phosphorylation promotes Nedd4 mediated K48-linked rather than K63-linked polyubiquitination of p38αV1 and p38αV2. Immunoblot analysis of anti-Flag immunoprecipitates of lysates of 293T cells co-transfected with various molecules (above lanes), probed with anti-HA (**A**,**B**). For whole cell lysates, p38αV1 and p38αV2 were probed with anti-Flag, and Nedd4 was probed with anti-Nedd4.

As indicated above, p38α might be a substrate of NEDD4 E3 ligase. Thus we speculated that Nedd4 might be responsible for the polyubiquitination of p38α and that phosphorylation of p38α might be involved in the biological course. To confirm the hypothesis, we began by co-transfecting p38αV1-Flag, Ubiquitin-HA, HA-Nedd4, MKK6-HA into 293T cells to detect p38αV1 ubiquitination. The results showed that Nedd4 might mediate K48 and K63-linked polyubiquitination of p38αV1 and that phosphorylation of p38α was involved in its ubiquitination (Fig. 5). To further confirm these results, we co-transfected p38αV1-Flag, K48-Ubiquitin-HA (or K63-Ubquitin-HA, whose K48 or K63 site is kept normal and other lysines of Ub are mutated and could not be conjugated to Ub by E3 ligase), HA-Nedd4, MKK6-HA into 293T cells to detect the ubiquitination types. From Fig. 6A, we can see that Nedd4 promoted K48-linked polyubiquitination of p38αV1 (lane 4 vs. lane 1) and K63-linked polyubiquitination of p38αV1 (lane 8 vs lane 5). Furthermore, Nedd4 promoted K48-linked polyubiquitination of p-p38αV1, (Fig. 6A, lane 3 vs. lane 2) rather than K63-linked polyubiquitination of p-p38αV1 (Fig. 6A, lane 7 vs. lane 6). In other words, the phosphorylation of p38αV1 promoted K48-linked polyubiquitination of p38αV1 by Nedd4 (Fig. 6A, lane 3 vs. lane 4) rather than K63-linked polyubiquitination of p38αV1 (Fig. 6A, lane 7 vs lane 8). These results led us to make the conclusion that Nedd4 probably promoted K48-linked and K63-linked polyubiquitination of p38αV1 and that the phosphorylation of p38αV1 by MKK6 promoted K48-linked rather than K63-linked polyubiquitination of p38αV1 by Nedd4.

Since the interaction between Nedd4 and p38αV2 is independent on the phosphorylation of p38αV2, thus we speculated that the polyubiquitination of p38αV2 by Nedd4 was different from that of p38αV1. We co-transfected p38αV2-Flag with other plasmids (as above) into 293T cells to detect p38αV2 ubiquitination. The results (Fig. 5, Right) showed that phosphorylation of p38αV2 was also involved in K48 and K63-linked polyubiquitination of p38αV2 and that Nedd4 promoted K48 and K63-linked polyubiquitination of p-p38αV2. To further confirm these results, we co-transfected p38αV2-Flag, K48-Ubiquitin-HA (or K63Ubquitin-HA), HA-Nedd4, MKK6-HA into 293T cells to detect the ubiquitination types. From Fig. 6B, we can see that phosphorylation of p38αV2 by MKK6 indeed enhanced K48-linked rather than K63-linked polyubiquitination of p38αV2 by Nedd4. As Fig. 6B indicated, Nedd4 promoted K48-linked polyubiquitination of p38αV2 (Fig. 6B, lane 4 vs. lane 1) and K63-linked polyubiquitination of p38αV2 (Fig. 6B, lane 8 vs. lane 5). Furthermore, Nedd4 promoted K48-linked (Fig. 6B, lane 3 vs. lane 2) rather than K63-linked polyubiquitination of p-p38αV2 by MKK6 (Fig. 6B, lane 7 vs. lane 6). In other words, the phosphorylation of p38αV2 by MKK6 promoted K48-linked polyubiquitination of p38αV2 by Nedd4 (Fig. 6B, lane 3 vs. lane 4) rather than K63-linked polyubiquitination of p38αV2 (Fig. 6B, lane 7 vs. lane 8). Thus, we came to the conclusion that phosphorylation of p38αV2 was involved in K48 and K63-linked polyubiquitination of p38αV2 and Nedd4 promoted K48-linked rather than K63-linked polyubiquitination of p-p38αV2.

As above, we could see that K48-linked and K63-linked polyubiquitination of p38α seems to be prevalent in the cell and K48-linked and K63-linked polyubiquitination might be respectively responsible for proteasomal and lysosomal degradation of p38α. However, it should be emphasized that there might be other types of ubiquitination besides K48 and K63-linked polyubiquitination of p38α by Nedd4 since there are branched chains in polyubiquitination.

### Nedd4 mediates degradation of p38αV1 and p38αV2

**Figure 7:**
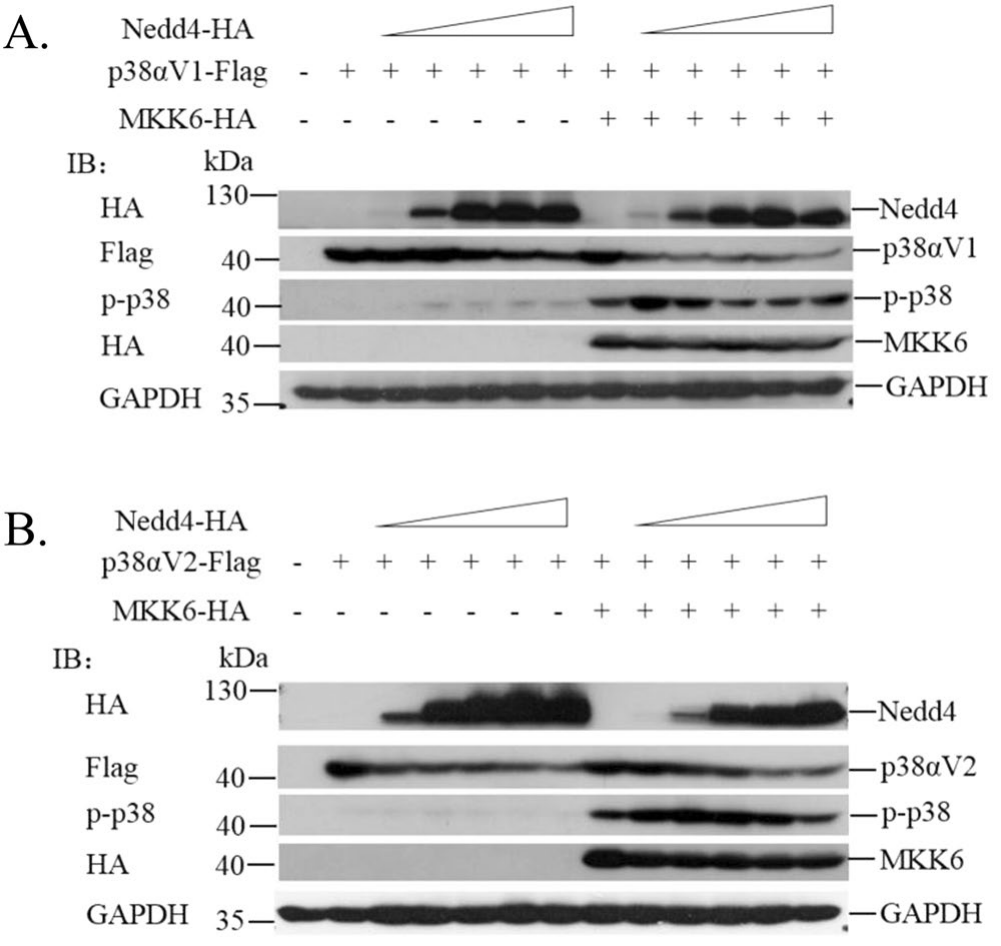
Nedd4 promotes degradation of p38αV1 and p38αV2. Immunoblots analysis of lysates of 293T cells co-transfected equivalent amounts of p38αV1-Flag (**A**) or p38αV2-Flag (**B**) and MKK6-HA plus increasing amounts of HA-Nedd4. Meanwhile, pcDNA3.1(+) empty vector was being added to ensure the same total amount of plasmids in each sample (**A**,**B**).

The effects of polyubiquitination on endogenous p-p38α were confirmed in iBMDM cells treated with the proteasome inhibitor MG132 or lysosome inhibitor Chloroquine treatment. Both MG132 and Chloroquine caused accumulation of p-p38α in iBMDM cells (Fig. 2B). Analysis of Nedd4 deficient cells indicated that polyubiquitination of p38α mediated by Nedd4 affected the level of p38α, especially p-p38α in response to LPS treatment (Fig. 1). To further investigate whether Nedd4 was responsible for the degradation of p38αV1 and p38αV2, we co-transfected 293T cells with p38αV1-Flag or p38αV2-Flag, MKK6-HA and increasing amounts of HA-Nedd4. Meanwhile, we added CHX (20 μg/ml)^27^ into cells to control total expressions of p38α protein. There seemed to be a dose-dependent effect of Nedd4 on the degradation of total p38αV1 in the absence of MKK6 (Fig. 7A, left). As speculated, Nedd4 might promote degradation of p-p38αV1 and total p38αV1 in a dose-dependent manner in the presence of MKK6 (Fig. 7A, right). From Fig. 7B, we could also see that both p-p38αV2 and total p38αV2 were degraded in a dose-dependent manner by Nedd4, whether there was MKK6 or not. However, there was a strange phenomenon that not all p38αV1 and p38αV2 were phosphorylated when there is too much empty pcDNA3(+) (Fig. 7A and B, lane 8) which might be resulted from interference of G418 resistant protein expressed by empty pcDNA3(+). Furthermore, Nedd4 mutant (Nedd4C774S) lost its ability to degrade p38αV1 and p38αV2 (data not shown), which indicated that the E3 ligase activity of Nedd4 is required for degradation. These results demonstrated that Nedd4 is responsible for the degradation of p38αV1 and p38αV2, and phosphorylation of p38αV1 and p38αV2 can promote their degradation by Nedd4.

### Multiple K residues of p38α are polyubiquitination sites by Nedd4

**Figure 8:**
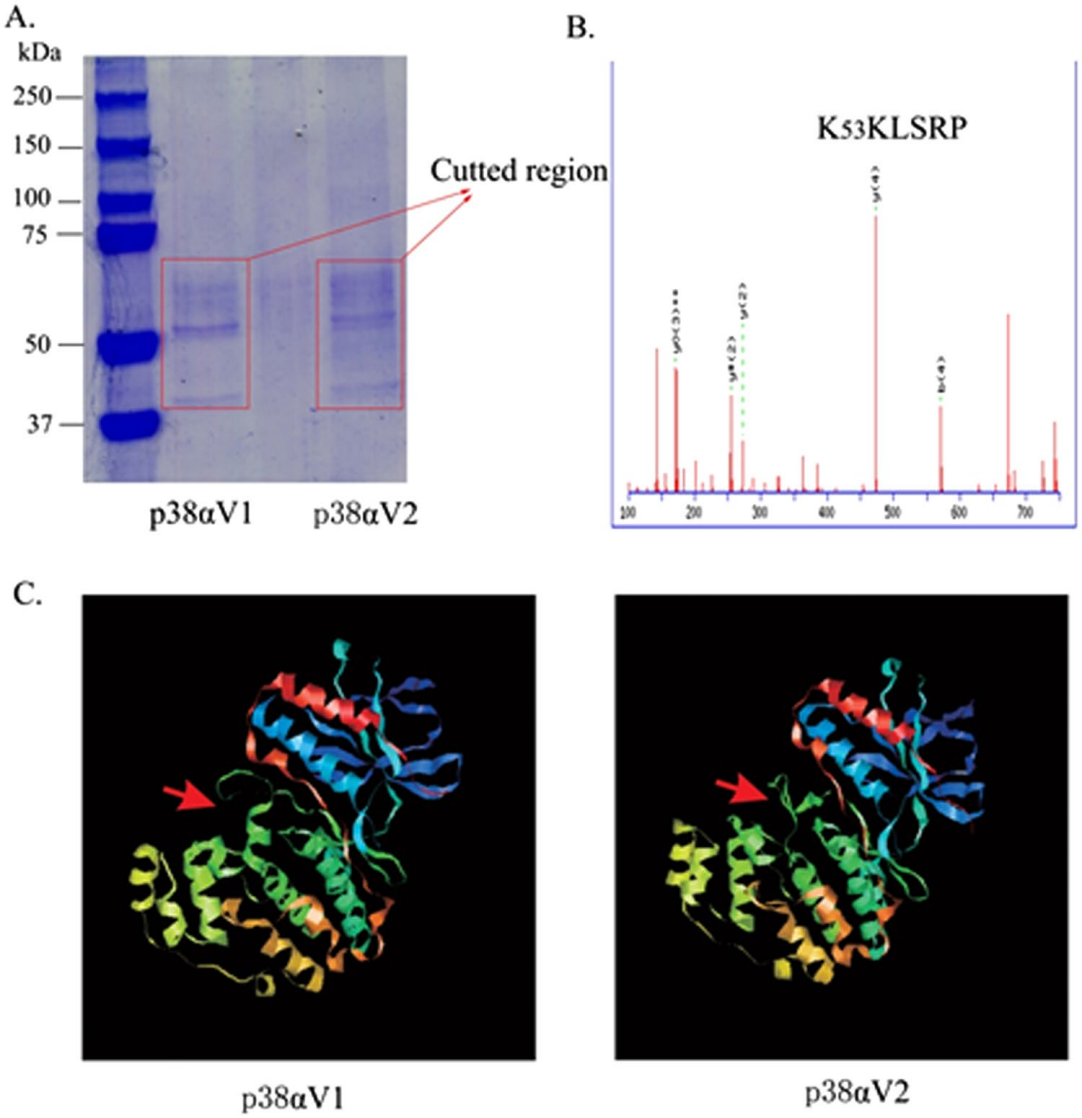
Analysis for polyubiquitination sites of p38α and conformation of p38αV1 and p38αV2. (**A**) Coomassie blue stained gel indicates the bands of p38αV1 and p38αV2 with polyubiquitination. 293T cells co-transfected with p38αV1-flag or p38αV2-flag, HA-Nedd4, MKK6-HA and ubiquitin-His were lysed, immunopricipitated with Ni-column and anti-Flag purification beads, gel-separated, and stained with Coomassie blue. The square area indicates the presence of ubiquinated-p38αV1 and p38αV2. (**B**) K53(K54) residue of p38α was identified as an common ubiquitination site in p38αV1 and p38αV2 by mass spectrometry. The protein sample with ubiquitinated p38αV1 and p38αV2 were recovered from the gel, enzyme digested, and subjected to mass analysis. Representative MS/MS spectra of peptides demonstrated ubiquitination at K53(K54) of p38αV1 and p38αV2. Peak matching expected y ions is labeled. (**C**) The conformational difference between p38αV1 and p38αV2. PDB documents (model No. 1p38 for p38αV1; model No. 3py3 for p38αV2) from SWISS-model, red arrows indicate the conformational difference between p38αV1 and p38αV2.

Although there was no ubiquitination site with high confidence on the protein sequences of p38αV1 or p38αV2 using online ubiquitination sites tool (http://www.ubpred.org/), we did find the polyubiquitination of p38αV1 and p38αV2 by Nedd4. Thus, Flag-tagged p38αV1 (or p38αV2), Nedd4-HA (or not), MKK6-HA and His-HA-ubiquitin were co-expressed in 293T cells to force polyubiquitination. After two-step immunoprecipitating ubiquitinated p38α with Ni-column and anti-Flag purification beads, the samples were separated by SDS-PAGE and visualized by Coomassie blue (Fig. 8A). In order to analyze ubiquitinated p38α by MS, the gel region containing ubiquitin-modified protein was excised, digested with trypsin, and analyzed by LC-MS (Thermo). A database search of the MS/MS spectra revealed that p38α was the predominant protein identified in the samples. There was no any ubiquitinated lysine in p38αV1 or p38αV2 when Nedd4 was not added (data not shown). Representative spectra demonstrated the K45, K53(K54), and K152 residues of p38αV1, and K53(K54), K287 and K295 residues of p38αV2 were identified to be modified with ubiquitin. The results showed that only K53(K54) residue was the common ubiquitination site between p38αV1 and p38αV2 (Fig. 8B) though all ubiquitination sites of p38αV1 and p38αV2 were in the conserved (or common) regions (Supplementary Fig. 3). We speculated that the different coverage rates of mass analysis might be one of reasons of different ubiquitinated sites between p38αV1 and p38αV2. Except that, we found that the stereochemical structures of p38αV1 and p38αV2 were different (Fig. 8C), which might be a major reason of different ubiquitinated sites between p38αV1 and p38αV2.

## Discussion

p38α plays an important role in such inflammatory diseases as skin inflammation, endotoxic shock and arthritis. Ubiquitination is an important posttranslational modification of proteins and plays a crucial regulatory role in inflammatory cells. However, p38α ubiquitination has never been reported. In this paper, we showed that E3 ubiquitin ligase Nedd4 is responsible for polyubiquitination and degradation of p38α through K48-linked and K63-linked polyubiquitination.

According to expressing characteristics of four p38 isoforms and the antibodies specific to p-p38 and total p38 used in experiments, we believed that the p-p38 and total p38 should be p-p38α and total p38α. Furthermore, we found that there were four variants of p38α, p38αV1 and p38αV2 expressed in full-length and the others were deficient in N terminal (Supplementary Fig. 3), which is why we came to the conclusion that the p-p38 and total p38 in iBMDM cells should be p38αV1 and p38αV2. The p-p38α was significantly increased in Nedd4 deficient iBMDM cells compared with total p38α responsive to LPS stimulation (Fig. 1), which conformed with the results that p-p38αV1 was more significantly decreased following increasing amounts of Nedd4 compared with total p38αV1 (Fig. 7). Furthermore, p-p38αV2 and total p38αV2 were gradually but significantly decreased following increasing amounts of Nedd4 (Fig. 7B). Thus we speculated that p38αV1 might be dominant in BMDM cells compared with p38αV2 in iBMDM cells. The result that p38α was not further increased in Nedd4^−/−^ iBMDM cells responsive to LPS at 60 min (Fig. 1D) might be related to the dephosphorylation of compensatory phosphatase for the p-p38α.

In general, the interaction between Nedd4 and its substrates is dependent on the interaction between the WW domain of Nedd4 and PPxY motif in the protein. Thus there is the possibility of interactions between Nedd4 and p38αV2 which contains PPaY motif. We did not detect the interaction between Nedd4 and p38αV1 in the absence of no MKK6, which might be due to the weak interaction between them. Phosphorylation of p38αV1 could enhance the interaction between Nedd4 and p38αV1, which seems to support that the WW domain of Nedd4 can also interact with phosphor-serine/threonine residues^18^. The polyubiquitination of p38αV1 by Nedd4 when there was no MKK further illustrates that there is a weak interaction between Nedd4 and p38αV1. From the data, we speculated the interaction between Nedd4 and p38αV2 is stronger than that between Nedd4 and p38αV1.

p38α phosphorylation status is able to modulate the level of polyubiquitination, which is line with status between phosphorylation and polyubiquitination of TAK1^28^. By adding MKK6 which is responsible for phosphorylation of p38α, the K48-linked and K63-linked polyubiquitination of p38αV1 and p38αV2 were increased (Figs 6 and 7). These results revealed a positive feedback loop for phosphorylation and polyubiquitination of p38αV1 and p38αv2, and indicated the dynamic regulation of p38α polyubiquitination and phosphorylation activation. It was reported that dual-site phosphorylation of p38α on T180/Y182 by MKK3/6 will result in conformation change, which might affect the type of polyubiquitination of p38α caused by Nedd4. The exact correlation needs to be investigated. However, we also found that the K48-linked and K63-linked polyubiquitination of p38αV1 by Nedd4 were increased compared with controls in the absence of MKK6 (Figs 5 and 6). Thus we speculated that the degradation of polyubiquitination of p38αV1 by Nedd4 could not counteract total p38αV1. The results of Fig. 7A showed that the total p38αV1 was indeed mildly decreased following increasing amounts of Nedd4 when MKK6 was not added, which offered evidence for our speculation.

TNF-α is one of the targets of p38α, so p38α activation (phosphorylation) will result in TNF-α mRNA and protein expression. Nedd4 silenced or knocked out in iBMDM cells leads to more phosphorylated p38α and subsequent more TNF-α expression in response to LPS stimulation. It was reported that another E3 ubiquitin ligase Itch bound directly to the TGF-β–activated kinase 1–binding protein 1 (Tab1) through a conserved PPXY motif and thus inhibited the activation of p38α^29^. Itch^−/−^ mice causes a skin-scratching phenotype and the mRNAs of pro-inflammatory cytokines, including TNF, IL-6, IL-1b, IL-11, and IL-19, were increased in the skin of Itch^−/−^ mice compared with that of wild-type mice. Though both Nedd4 and Itch are HECT E3 ubiquitin ligases, they negatively regulate TNF-α production by different mechanisms in macrophages. However, RING E3 ubiquitin ligase Cbl-b, which is one of the substrates of Nedd4, was reported to inhibit TLR signaling by degrading MyD88 and TRIF^30^. Nedd4 deficiency resulted in enhancement of Cbl-b (data not shown) that would also inhibit TLR response according to the paper. The discrepancy with our results might arise from the fine regulation mechanism of the cell through other complement signaling. Thus the function of Nedd4 in inflammatory diseases calls for further investigation through conditional knockout nedd4 mice.

The discrepancy of the interaction and polyubiquitination sites between p38αV1 and p38αV2 might result from the stereochemical structure caused by different amino acids. Furthermore, since the coverage rates of mass analysis between p38αV1 and p38αV2 were different, the polyubiquitination sites of p38αV1 and p38αV2 obtained by mass analysis need to be further confirmed by detecting ubiquitination of p38αV1 and p38αV2 with different K sites mutation by Nedd4 in the future.

In summary, our study demonstrates that E3 ubiquitin ligase Nedd4 is responsible for polyubiquitination of p38α with multiple K sites. Furthermore, Nedd4 induces degradation of p38α was in a dose-dependent manner. Thus we have revealed a new regulatory mechanism in innate immune cells that might be exploited therapeutically in p38α activation-induced inflammatory disorders.

## Materials and Methods

### Reagents and materials

The following commercial antibodies were used: HRP-conjugated anti-mouse IgG (sc2005), HRP-conjugated anti-rabbit IgG (sc2004), HRP-conjugated anti-rat IgG (sc2006) and NEDD4-1 (H-135) Rabbit polyclonal IgG (sc25508) were from Santa cruz. VeriBlot for IP secondary antibody-HRP (ab131366) was from Abcam. K48-linkage specific polyubiquitin (D905) Rabbit mAb (8081 S) and K63-linkage specific polyubiquitin (D7A11) Rabbit mAb (5621 S), p-P38 MAPK (T180/Y182) (D3F9) Rabbit mAb (4511 S), and p38 MAPK (D13E1) Rabbit mAb from CST, GAPDH rabbit IgG from Proteintech, Anti-HA antibody produced in rabbit (H6908-2ML) from Sigma, and Flag-Tag mouse mAb (KM8002) from SanJian (Tianjin, China). Anti-flag M2 affinity Gel (A2220), 011:B4 LPS, Cycloheximide (CHX), MG132 (C2211) and chloroquine (C6628) were obtained from Sigma. Protease inhibitors cocktail (539134) was purchased from Calbiochem. Murine TNF-α (Mini EDK) ELISA kit (900-M54) was from PeproTECH. TransStart Top Green qPCR SuperMix (K20508) was from Transgen (Beijing, China). NI-NTA superflowsepharose was from CMCTag (P3403). Sequencing-grade trypsin was purchased from Promega (0.5 mg/ml).

### Cell culture and treatment

Human 293T was obtained from American Type Culture Collection (Manassas, VA). C57BL/6 mice-derived iBMDM cells were gift from Feng Shao (NIBS)^31^. The cells were grown in Dulbecco’s modified Eagle’s medium (DMEM) containing 10% 56 °C complement inactivated fetal bovine serum (FBS), and 1% (v/v) penicillin-streptomycin solution. For ligand stimulation, cells were washed once with PBS, and incubated with 100 ng/mL LPS at indicated times.

### Plasmids

The lentiviral vector expressing shRNA specific to Nedd4 was a gift from Hongrui Wang (Xiamen University). pcDNA3.1(−)-HA-Nedd4/2795 was gift from Allan Weissman (Addgene plasmid#11426)^32^. pcDNA3.1-Ub-HA, pcDNA3.1-UbK48-HA, pcDNA3.1-UbK63-HA, were kindly provided by Lingqiang Zhang (Beijing Institute of Radiation Medicine) for *in vivo* ubiquitination assay. The lentivirus packaging plasmids, pLp1, pLp2 and VsVG were from Invitrogen. The pcDNA3.1(+)-p38αV1-Flag, pcDNA3.1(+)-p38αV2-Flag plasmids were constructed by ourselves. p38α cDNA was obtained by overlapping PCR using the following primers: p38α-F: 5′-CTAGCTAGCATGTCGCAGGAGAGGCCCACGTT-3′, p38αR1: 5′-CGGGATCCCTACTTGTCGTCATCGTCTTTGTA-3′, p38α-R2: 5′GTCATCGTCTTTGTAGTCGGACTCCATTTCTTCTTGGTC-3′. The p38αV2 identifying primers are as follows: 5′-ATGTCGCAGGAGAGGCCCAC-3′ and 5′-CTCATGGCTTGGCATCCTGT-3′. All vectors were confirmed by DNA sequencing.

### Lentivirus production and infection

Lentiviral packaging was as previously described^33^. The 293 T cells were transfected with the lentivirus-based constructs (shCon and shNedd4) along with packaging plasmids, pLp1, pLp2 and VsVG by using Ca_3_(PO4)_2_ precipitation method. Virus-containing medium was collected at 48 hours, and 72 hours post-transfection. iBMDMs cells were infected with lentiviral-containing medium in the presence of 8 μg/ml polybrene (Sigma). After 24 hours, the virus-containing medium was replaced with selection medium containing 5 mg/ml G418 (Invivo). After cell growth was stable, cells were used in subsequent experiments.

### Nedd4^−/−^ iBMDM construction

The nedd4^−/−^ iBMDM cell line was derived from limited dilution of iBMDM cells which were electotransfected with PX330 plasmid coding a quide RNA specific to exon 1 of Nedd4 genome and Cas9. The 20nt guide sequence target for Nedd4 gene possesses the highest score in those guide sequences specific to 29 exons of Nedd4 gene designed through an online CRISPR Design Tool (http://tools.genomeengineering.org) which provided by Feng Zhang’s group^34^. The single strand DNA (ssDNA) sequences were g1F: 5′-caccgTACTGGGGCCTCCGACTCGT-3′ and g1R: 5′-cATGACCCCGGAGGCTGAGCAcaaa-3′ and were synthesized by Life Invitrogen. The double strand DNA was formed with the mixed ssDNA by gradually cooling from 100 °C to 4 °C, which was subsequently ligated with PX330 fragment digested with BbsI (FD1014, Thermo). Then, the ligated plasmid was transformed into the competent DH5α. The sequence of the vector was confirmed by sequence, and the correct vector was electroporated into iBMDM cells using the NeonTransfection system (ThermoFisher) following the manufacturer’s instructions.

Nedd4 KO-1 and Nedd4 KO-2 iBMDM cell lines were established through puromycin selection after being transfected with PX330 containing sequences coding a guide RNA specific to exon 1 and exon 4 of Nedd4 genome respectively and Tia1l plasmid^35^ inserted with the sequence including porcine 2A, anti-puromycin, terminal codon and polyA tail.

### Immunoblotting

For protein interaction assays, cells were lysed with 0.5% NP40 buffer. For ubiquitination assays, cells were lysed in ice-cold RIPA lysis buffer containing 50 mM Tris-HCl, pH 8.0, 150 mM NaCl, 1% Nonidet P-40, 0.5% deoxycholate, 0.1% SDS, and protease inhibitors cocktail. After centrifugation, cell extracts were resolved by SDS-PAGE and analyzed by immunoblotting. The membranes were probed with the indicated antibodies. Blots were visualized by Clarity^Tm^ western ECL substrate exposed on X-ray film (Kodak film). For densitometric quantification, the Western blot images were analyzed with ImageJ 1.48 (Windows version of NIH Image, http://rsb.info.nih.gov/nih-image/). The relative amount was calculated and the band with the highest intensity was set as 1. The data was presented as mean values from duplicated experiments.

### Immunoprecipitation

Cell lysates were prepared by ice-cold lysis buffer, and immunoprecipitated with the indicated antibodies in immunoprecipitation buffer containing 10 mM Tris-HCl, pH 7.0, 150 mM NaCl, and 0.5% NP-40 4 h at 4 °C and protease inhibitors (1:500). All samples were incubated with protein A/G–Sepharose (SantaCruz) overnight at 4 °C. The beads were washed three times with ice-cold lysis buffer or immunoprecipitation buffer, separated by SDS-PAGE and analyzed by immunoblotting with indicated antibodies. Blots were developed with chemiluminescence reagent.

### *In vivo* ubiquitination assays

Flag-tagged protein and HA-tagged ubiquitin were co-expressed in 293T cells. 24 hours post-transfection, MG132 (10 μM) and Chloroquine (40 μM) were added for 8 h, then the cells were lysed with harsh RIPA lysis buffer containing protease inhibitors (1:500), and 1% SDS on ice for 1 h, and denatured by heating for 5 min. Ultrasonicated 3 times with dr.hielscher (up200s) (2 min/time, 0.5 × 50%), supernatants were diluted with lysis buffer until the concentration of SDS was decreased to 0.1%, followed by reimmunoprecipitation with the appropriate antibodies. The lysate was immunoprecipitated with Flag antibody overnight at 4 °C, and pulled-down with protein A/G beads. The samples were washed three times with RIPA lysis buffer, gel-separated and analyzed by immunoblotting. Immunoprecipitates were analyzed by immunoblot with anti-ubiquitin or antibody specific for K48/K63-linked ubiquitin or anti-HA.

### Real-time quantitative polymerase chain reaction

Total cellular RNA from 1 × 10^5^ cells was isolated with TRIzol (Invitrogen), and used to synthesize first-strand cDNA with cDNA synthesis kit (Thermo, Fermentas). mRNA amounts were quantified by real-time quantitative polymerase chain reaction (RT-qPCR) with the TransStart Green qPCR SuperMix. Primer sequences are as follows, Nedd4-F: 5′-GTACTCTCGGAGGACGAGGT-3, Nedd4-R: 5′-CGTAAGGATCACTGGCTCCC-3′, TNF-α-F: 5′-CTGTGAAGGGAATGGGTGTT-3′, TNF-α-R: 5′-CAGGGAAGAATCTGGAAAGGTC-3′, GAPDH-F: 5′-ATCATCTCCGCCCCTTCTGC-3′, GAPDH-R: 5′-CCATCACGCCACAGCTTTCC-3′. All values were normalized to the level of GAPDH mRNA expression.

### The enzyme-linked immunosorbent assay (ELISA)

Cells (3 × 10^5^) were seeded in a 12-well culture plate overnight. After stimulation with the indicated ligands for indicated times, the cytokine-containing medium was collected, and the protein level was measured with mouse TNF-α kit.

### Luciferase reporter assay

The procedure in luciferase reporter assays was described before^33^. 293T cells were seeded into 24-well plate, and co-transfected with a mixture of MyD88-HA plasmid, pGL3-AP-1 reporter, pGL3-TK reporter and various amounts of Nedd4-HA for 24 h using lipofactamine 3000 (Invitrogen). The luciferase activity was measured with Dual-Glo luciferase assay system (Promega) following the manufacturer’s instructions. Luciferase activity was normalized using the Renilla luciferase activity.

### Sample preparation for mass analysis

Flag-tagged p38αV1 (or p38αV2), His-HA-tagged ubiquitin and HA-tagged Nedd4 were co-expressed in 293T cells. After 24 hours, cells were collected and lysated with 700 ul LE buffer (50 mM NaH_2_PO4, 300 mM NaCl, pH = 8.0). After being sonicated with dr.hielscher (up 200 s) for 3 times (2 min/time, 0.5 × 50%), the cell lysis was diluted to 3 ml with LE buffer. Then 600ul NI-NTA superflowsepharose was added for incubation at 4 °C overnight. The Ni agrose was washed with washing buffer (50 mM NaH_2_PO4, 300 mM NaCl, 10 mM imidazole, pH = 8.0) for 5 times. The ubiquitinated proteins were eluted with 7 ml elution buffer (50 mM NaH_2_PO4, 300 mM NaCl, 250 mM imidazole, pH = 8.0). The solution was replaced by 3 kDa concentration tube (Millipore) with 7 ml lysis buffer (Sigma). Then the protein solution was incubated with 100 µ l Anti-flag M2 affinity Gel (Sigma) at 4 °C overnight. Samples were gel-separated, and stained with Coomassie blue. Protein samples containing ubiquinated-p38αV1 and p38αV2 were recovered from the gel, and subjected to enzyme digestion according to the following procedure. The gel was cut into 1 mm^3^ particles and decolorized with 50% ACN 25 mM NH_4_HCO_3_ and 10 mM DTT at 56 °C for 1 h. After being dissolved with 55 mM IAA at room temperature in the dark, freeze-dried proteins were digested with trypsin solution (15 ng/µL) at 4 °C for 1 h, then 25 mM NH_4_HCO_3_ was added over gel particles at 37 °C for 16 h. 5% TFA and 2.5% TFA/50% ACN was successively added at 37 °C for 1 h to extract peptides. The extracts were freeze-dried and dissolved with mobile phase A (2% ACN + 98% H_2_O + 0.1% TFA). After being centrifuged at 15,000 rpm for 15 min, the supernatant was used for mass analysis.

### Liquid chromatography-mass spectrometry

Mass spectral data were acquired using a Thermo Q-Exactive mass spectrometer. 20 µL of samples were injected on to a Easy nLC 1000 column (Thermo Scientific)(150 µm I.D. × 1.9 µm, 12 cm) trapped with column (100 µm I.D. × 3 µm, 2 cm) using 0.1% formic acid in water as mobile phase A and 0.1% formic acid in 80% acetonitrile as mobile phase B operated at the flow rate of 600 nL/min. Separation was attained using a solvent gradient ranging from 10% to 100% of mobile phase B in 70 min. The mass scanning range was set from m/z 300 to 1,400 (Resolution as 7,000, AGC target as 3 × 10^6^, Maximum as 60 ms) for the survey full-scan MS mode, and dd-MS^2^/dd-SIM was set as following parameters (Resolution as 17,500, AGC target as 5 × 10^4^, Maximum as 80ms, loop count as 20, Isolation window as 3.0 m/z, fixed first mass as 100 m/z, NCE/stepped Ac as 27). The dd setting was set as (underfill ratio as 1.0%, charge exclusion as 1, 7, 8, >8, peptide match as on, Exclude isotopes as on, dynamic exclusic as 18.0 ms). For data analysis, all MS/MS spectra were converted to mzXML and mgf format from the experimental RAW file using MM File Conversion Tools (http://www.massmatrix.net), and then analyzed by Mascot search. The search parameters in Mascot including the error tolerance of precursor ions and the MS/MS fragment ions in spectra were 6 ppm and the enzyme was assigned as trypsin with three missed cleavages allowed. The variable post-translational modifications in the search parameters were assigned to include the oxidation of methionine, carbamidomethylation of cysteine, and ubiquitination of lysine.

### Statistical analysis

Data are expressed as the mean ± SD. All statistical analysIs were conducted using SPSS 18.0. Student’s t test was used to assess the difference between controls and indicated groups. For all comparisons, a value of P < 0.05 was considered significant (two-tailed). Unless indicated, results were derived from three independent experiments with similar results. Bars indicated standard deviation.

## Electronic supplementary material

Supplementary Dataset 3
