# Supplementary material for: E3 ubiquitin ligase Nedd4 inhibits AP-1 activity and TNF-α production through targeting p38α for polyubiquitination and subsequent degradation
Source: Sci Rep. 2017 Jul 3;7:4521. doi: 10.1038/s41598-017-04072-2 (PMC5495757; doi:10.1038/s41598-017-04072-2)
Supplement: Supplementary file 1 — Supplementary Dataset 3 [file 41598_2017_4072_MOESM1_ESM.pdf]

E3 ubiquitin ligase Nedd4 inhibits AP-1 activity and TNF-α production through targeting p38α for polyubiquitination and subsequent degradation

Qingjun Liu[1*], Shihui Zhang[1], Gan Chen[1], Hong Zhou[1*]

1. Beijing Institute of Transfusion Medicine, Beijing Key Laboratory of Blood Safety and Supply Technologies, Taiping Road 27, 100850 Beijing, P.R.China

*: Corresponding author: qjliu2003@163.com or zhouhtt1966@163.com

Supplementary Data:

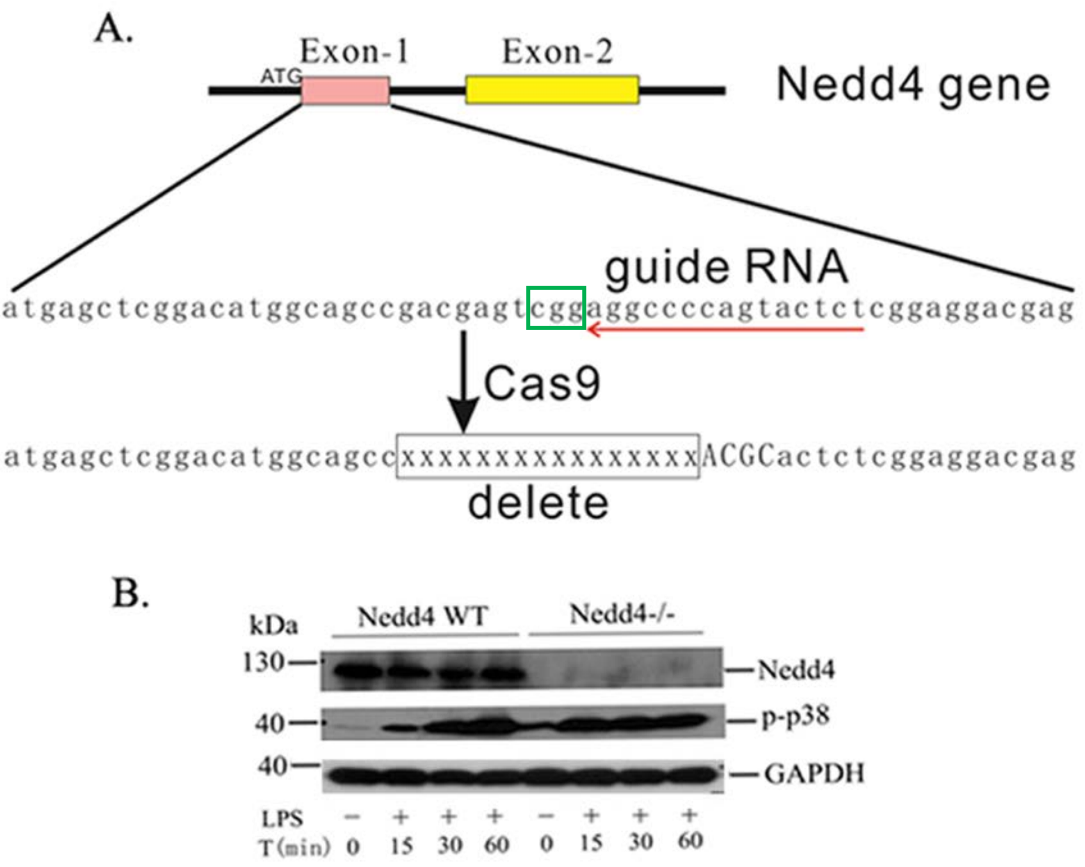

Supplement figure 1: A. Nedd4$^{-/-}$ iBMDM cells generated by CRISPR/Cas9-mediated targeting. iBMDM cells were electrotransfected with PX330 plasmid coding a small guide RNA (sgRNA) specific to exon1 of Nedd4 and Cas9, then were diluted by limited dilution. The clones were identified by Western Blot and sequencing. The red arrow indicates the target DNA sequence paired with the sgRNA. The green pane indicates the PAM motif adjacent to target DNA. A double strand break (DSB) induced by Cas9 was repaired by endogenous DNA repair machinery and rejoined, which resulted in 16 bases deletion and the creation of a premature stop codon resulting in Nedd4 gene knockout. **B. Immunoblot analysis of Nedd4$^{-/-}$ iBMDM cells.** The phosphorylated p38 protein levels are increased in Nedd4$^{-/-}$ iBMDM cells stimulated for 0-60 min (above lanes) with LPS.

```
                                                                    Section 8
           (379) 379        390        400        410        420         432
   p38-v1  (379) CCCGCTGCTGCCGCTGGAAGATGTCGCAGGAGAGGCCCACGTTCTACCGGCAGG
   p38-v2  (379) CCCGCTGCTGCCGCTGGAAGATGTCGCAGGAGAGGCCCACGTTCTACCGGCAGG
   p38-v3    (1) --------------------GCCTTGCAAG-GACGCTCCAGCTCGCCGCTTAGT
   p38-v4  (379) CCCGCTGCTGCCGCTGGAAGATGTCGCAGGAGAGGCCCACGTTCTACCGGCAGG
Consensus  (379) CCCGCTGCTGCCGCTGGAAGATGTCGCAGGAGAGGCCCACGTTCTACCGGCAGG
                                                                    Section 9
           (433) 433    440        450        460        470             486
   p38-v1  (433) AGCTGAACAAGAC-CATCTGGGAGGTGCCCGAACGATACCAGAACCTGTCCCCG
   p38-v2  (433) AGCTGAACAAGAC-CATCTGGGAGGTGCCCGAACGATACCAGAACCTGTCCCCG
   p38-v3   (34) CACATACCACTGCTCATTTCAGTATTGTTTGA-CAAAACAGTTTTCCATACCGA
   p38-v4  (433) AGCTGAACAAGAC-CATCTGGGAGGTGCCCGAACGATACCAGAACCTGTCCCCG
Consensus  (433) AGCTGAACAAGAC CATCTGGGAGGTGCCCGAACGATACCAGAACCTGTCCCCG
                                                                   Section 10
           (487) 487          500        510        520        530       540
   p38-v1  (486) GTGGGCTCGGGCGCCTATGGCTCGGTGTGT------------------------
   p38-v2  (486) GTGGGCTCGGGCGCCTATGGCTCGGTGTGT------------------------
   p38-v3   (87) GCAGAGGGGCGCCCCTCAAGATCAAGAAGT------------------------
   p38-v4  (486) GTGGGCTCGGGCGCCTATGGCTCGGTGTGTCCCTTAGAAGATTTGCGGAGTTTT
Consensus  (487) GTGGGCTCGGGCGCCTATGGCTCGGTGTGT
                                                                   Section 11
           (541) 541      550        560        570        580           594
   p38-v1  (516) ---------------------GCTGCTTTTGATACAAAGACGGGGCATCGTGTG
   p38-v2  (516) ---------------------GCTGCTTTTGATACAAAGACGGGGCATCGTGTG
   p38-v3  (117) ---------------------GCTGCTTTTGATACAAAGACGGGGCATCGTGTG
   p38-v4  (540) ATCAGCTTGTAAGTACCGAGTGCTGCTTTTGATACAAAGACGGGGCATCGTGTG
Consensus  (541)                      GCTGCTTTTGATACAAAGACGGGGCATCGTGTG
```

……

```
                                                                   Section 21
          (1081) 1081     1090       1100       1110       1120          1134
   p38-v1 (1035) ATGGCTGAGCTGTTGACCGGAAGAACGTTGTTTCCTGGTACAGACCATATTGAT
   p38-v2 (1035) ATGGCTGAGCTGTTGACCGGAAGAACGTTGTTTCCTGGTACAGACCATATTAAC
   p38-v3  (636) ATGGCTGAGCTGTTGACCGGAAGAACGTTGTTTCCTGGTACAGACCATATTGAT
   p38-v4 (1080) ATGGCTGAGCTGTTGACCGGAAGAACGTTGTTTCCTGGTACAGACCATATTGAT
Consensus (1081) ATGGCTGAGCTGTTGACCGGAAGAACGTTGTTTCCTGGTACAGACCATATTGAT

                                                                   Section 22
          (1135) 1135 1140       1150       1160       1170             1188
   p38-v1 (1089) CAGTTGAAGCTCATTTTAAGACTCGTTGGAACCCCAGGGGCTGAGCTTCTGAAG
   p38-v2 (1089) CAGCTTCAGCAGATAATGCGTCTGACGGGGACACCCCCTGCTTATCTCATTAAC
   p38-v3  (690) CAGTTGAAGCTCATTTTAAGACTCGTTGGAACCCCAGGGGCTGAGCTTCTGAAG
   p38-v4 (1134) CAGTTGAAGCTCATTTTAAGACTCGTTGGAACCCCAGGGGCTGAGCTTCTGAAG
Consensus (1135) CAGTTGAAGCTCATTTTAAGACTCGTTGGAACCCCAGGGGCTGAGCTTCTGAAG
                                                                   Section 23
          (1189) 1189        1200       1210       1220       1230       1242
   p38-v1 (1143) AAAATCTCCTCAGAGTCTGCAAGAAACTACATTCAGTCTCTGGCCCAGATGCCG
   p38-v2 (1143) AGGATGCCAAGCCATGAGGCAAGAAACTACATTCAGTCTCTGGCCCAGATGCCG
   p38-v3  (744) AAAATCTCCTCAGAGTCTGCAAGAAACTACATTCAGTCTCTGGCCCAGATGCCG
   p38-v4 (1188) AAAATCTCCTCAGAGTCTGCAAGAAACTACATTCAGTCTCTGGCCCAGATGCCG
Consensus (1189) AAAATCTCCTCAGAGTCTGCAAGAAACTACATTCAGTCTCTGGCCCAGATGCCG
```

Supplement figure 2. **Alignment of mouse p38α mRNA nucleotide variants.** At the first variant region, the sequence of nucleotide of p38αMAPK variant 1 (p38αV1) is same as that of p38αV2. The p38αV4 inserts 45 nucleotides and p38αV3 deletes 398

nucleotides compared with p38αV1 and p38αV2. However, the initial codon of p38αV3 and p38αV4 are at the behind of the first variant region (the first coding nucleotide of p38αV3 and p38αV4 are at 231 site and at 675 site respectively), thus the protein sequences of p38αV3 is same as that of p38αV4. At the second variant region, the p38αV2 contains different nucleotide compared with the sequences of p38αV1, V3 and V4.

```
                                                                            Section 1
                      (1) 1            10              20              30               49
p38-isoform1 human    (1) MSQERPTFYRQELNKTIWEVPERYQNLSPVGSGAYGSVCAAFDTKTGLR
       p38v2-mouse    (1) MSQERPTFYRQELNKTIWEVPERYQNLSPVGSGAYGSVCAAFDTKTGHR
       p38V1-mouse    (1) MSQERPTFYRQELNKTIWEVPERYQNLSPVGSGAYGSVCAAFDTKTGHR
       p38V3-mouse    (1) -------------------------------------------------
         Consensus    (1) MSQERPTFYRQELNKTIWEVPERYQNLSPVGSGAYGSVCAAFDTKTGHR
                                                                            Section 2
                     (50) 50              60              70              80               98
p38-isoform1 human   (50) VAVKKLSRPFQSIIHAKRTYRELRLLKHMKHENVIGLLDVFTPARSLEE
       p38v2-mouse   (50) VAVKKLSRPFQSIIHAKRTYRELRLLKHMKHENVIGLLDVFTPARSLEE
       p38V1-mouse   (50) VAVKKLSRPFQSIIHAKRTYRELRLLKHMKHENVIGLLDVFTPARSLEE
       p38V3-mouse    (1) ----------------------------MKHENVIGLLDVFTPARSLEE
         Consensus   (50) VAVKKLSRPFQSIIHAKRTYRELRLLKHMKHENVIGLLDVFTPARSLEE
                                                                            Section 3
                     (99) 99          110             120             130              147
p38-isoform1 human   (99) FNDVYLVTHLMGADLNNIVKCQKLTDDHVQFLIYQILRGLKYIHSADII
       p38v2-mouse   (99) FNDVYLVTHLMGADLNNIVKCQKLTDDHVQFLIYQILRGLKYIHSADII
       p38V1-mouse   (99) FNDVYLVTHLMGADLNNIVKCQKLTDDHVQFLIYQILRGLKYIHSADII
       p38V3-mouse   (22) FNDVYLVTHLMGADLNNIVKCQKLTDDHVQFLIYQILRGLKYIHSADII
         Consensus   (99) FNDVYLVTHLMGADLNNIVKCQKLTDDHVQFLIYQILRGLKYIHSADII
                                                                            Section 4
                    (148) 148         160             170             180              196
p38-isoform1 human  (148) HRDLKPSNLAVNEDCELKILDFGLARHTDDEMTGYVATRWYRAPEIMLN
       p38v2-mouse  (148) HRDLKPSNLAVNEDCELKILDFGLARHTDDEMTGYVATRWYRAPEIMLN
       p38V1-mouse  (148) HRDLKPSNLAVNEDCELKILDFGLARHTDDEMTGYVATRWYRAPEIMLN
       p38V3-mouse   (71) HRDLKPSNLAVNEDCELKILDFGLARHTDDEMTGYVATRWYRAPEIMLN
         Consensus  (148) HRDLKPSNLAVNEDCELKILDFGLARHTDDEMTGYVATRWYRAPEIMLN
                                                                            Section 5
                    (197) 197           210             220             230              245
p38-isoform1 human  (197) WMHYNQTVDIWSVGCIMAELLTGRTLFPGTDHINQLQQIMRLTGTPPAY
       p38v2-mouse  (197) WMHYNQTVDIWSVGCIMAELLTGRTLFPGTDHINQLQQIMRLTGTPPAY
       p38V1-mouse  (197) WMHYNQTVDIWSVGCIMAELLTGRTLFPGTDHIDQLKLILRLVGTPGAE
       p38V3-mouse  (120) WMHYNQTVDIWSVGCIMAELLTGRTLFPGTDHIDQLKLILRLVGTPGAE
         Consensus  (197) WMHYNQTVDIWSVGCIMAELLTGRTLFPGTDHINQLQQILRLVGTPPAY
                                                                            Section 6
                    (246) 246             260             270             280              294
p38-isoform1 human  (246) LINRMPSHEARNYIQSLTQMPKMNFANVFIGANPLAVDLLEKMLVLDSD
       p38v2-mouse  (246) LINRMPSHEARNYIQSLAQMPKMNFANVFIGANPLAVDLLEKMLVLDSD
       p38V1-mouse  (246) LLKKISSESARNYIQSLAQMPKMNFANVFIGANPLAVDLLEKMLVLDSD
       p38V3-mouse  (169) LLKKISSESARNYIQSLAQMPKMNFANVFIGANPLAVDLLEKMLVLDSD
         Consensus  (246) LINKISSHSARNYIQSLAQMPKMNFANVFIGANPLAVDLLEKMLVLDSD
                                                                            Section 7
                    (295) 295   300             310             320             330          343
p38-isoform1 human  (295) KRITAAQALAHAYFAQYHDPDDEPVADPYDQSFESRDLLIDEWKSLTYD
       p38v2-mouse  (295) KRITAAQALAHAYFAQYHDPDDEPVADPYDQSFESRDLLIDEWKSLTYD
       p38V1-mouse  (295) KRITAAQALAHAYFAQYHDPDDEPVADPYDQSFESRDLLIDEWKSLTYD
       p38V3-mouse  (218) KRITAAQALAHAYFAQYHDPDDEPVADPYDQSFESRDLLIDEWKSLTYD
         Consensus  (295) KRITAAQALAHAYFAQYHDPDDEPVADPYDQSFESRDLLIDEWKSLTYD
                                                                            Section 8
                    (344) 344     350           360
p38-isoform1 human  (344) EVISFVPPPLDQEEMES
       p38v2-mouse  (344) EVISFVPPPLDQEEMES
       p38V1-mouse  (344) EVISFVPPPLDQEEMES
       p38V3-mouse  (267) EVISFVPPPLDQEEMES
         Consensus  (344) EVISFVPPPLDQEEMES
```

Supplement figure 3. Alignment of p38α protein variants. Both p38αV3 and p38αV4 lack 77 N terminal amino acids of mouse p38αV1 and p38αV2. The protein sequence of p38αV1 is same as p38αV3 at the second variant region. The protein sequence of

mouse p38αV2 is same as that of human p38αV1 and both of them contain PPAY motif to which Nedd4 binds. The Red pane indicates PPAY motif.
